# Intrathecal Ropivacaine Versus Levobupivacaine for Infraumbilical Surgery: A Randomized Double-Blind Trial

**DOI:** 10.7759/cureus.113255

**Published:** 2026-07-23

**Authors:** J.G. Jennifer Lydia, Arthi Asokan, Iswaryarajan Hercule M.S, Sivaperumal G, Arunkumar Muthalu, Vimala Ananthy

**Affiliations:** 1 Anesthesiology and Critical Care, Porunai Hospitals, Tirunelveli, IND; 2 Anesthesiology, Sri Venkateshwaraa Medical College Hospital and Research Centre, Puducherry, IND; 3 Anesthesiology, Meenakshi Mission Hospital and Research Centre, Madurai, IND; 4 Anesthesiology, Aarupadai Veedu Medical College, Puducherry, IND; 5 Critical Care, Mahatma Gandhi Medical College and Research Institute, Puducherry, IND; 6 Pharmacology, Mahatma Gandhi Medical College and Research Institute, Sri Balaji Vidyapeeth (Deemed to be University), Puducherry, IND

**Keywords:** analgesia, anesthesia neurotoxicity, cardiotoxicity, infraumbilical surgery, intrathecal, levobupivacaine, local anesthetic agent, lower abdomen, lower limb surgery, ropivacaine

## Abstract

Background

Newer-generation amide local anesthetic drugs are relatively less cardiotoxic and neurotoxic than bupivacaine and thus can be used as safer alternatives to reduce bupivacaine-related toxicity. In the present study, we hypothesized that isobaric 0.5% levobupivacaine would be more efficacious than 0.75% ropivacaine in terms of the onset and duration of sensory and motor block, hemodynamic effects, and duration of analgesia.

Methods

Ninety patients who met the inclusion criteria were enrolled and randomly assigned to one of two groups. Patients in Group L received 3 mL of 0.5% levobupivacaine (n = 45), and those in Group R received 3 mL of 0.75% ropivacaine (n = 44). Evaluations were performed at different time intervals to assess sensory and motor block, hemodynamic parameters, analgesic effectiveness, duration of analgesia, side effects, Visual Analog Scale score, and the need for rescue analgesia.

Results

Patients in Group L experienced a faster onset of sensory block (5.9 minutes) and motor block (8.6 minutes) than patients in Group R, who had a sensory block onset of 8.3 minutes and a motor block onset of 11.7 minutes. The duration of sensory block was longer in Group L, with a mean duration of 268 minutes, compared with Group R, which had a mean duration of 160 minutes. The duration of motor block was also longer in Group L (230 minutes) than in Group R (136 minutes) (p < 0.0001).

Conclusions

Both ropivacaine and levobupivacaine provided adequate subarachnoid block for infraumbilical surgeries. However, levobupivacaine provided more effective anesthesia, with a faster onset, longer duration of sensory and motor block, and prolonged postoperative analgesia compared with ropivacaine. The prolonged sensory block with levobupivacaine resulted in less postoperative pain and reduced the need for early rescue analgesia, but at the cost of prolonged motor blockade. Both drugs are more cardiostable than bupivacaine and can be used safely as alternatives to bupivacaine.

## Introduction

Spinal anesthesia is the most frequently utilized regional anesthesia technique for surgeries involving the infraumbilical region. It is an economical, dependable, and safe method that provides both surgical anesthesia and prolonged postoperative pain relief [1]. By enabling a more rapid onset and effective sensory and motor blockade, spinal anesthesia reduces intraoperative pain as well as endocrine, motor, and autonomic responses. Spinal anesthesia is favored because it is easier to administer, requires less time to perform, and has a higher success rate than other regional blocks using either blind or ultrasound-guided techniques. Although bupivacaine is the most commonly used local anesthetic for this purpose, it has the disadvantage of cardiotoxicity because of the R isomer [2]. Two newer amide local anesthetics, ropivacaine and levobupivacaine, are currently used to reduce the toxicity associated with bupivacaine because of their lower cardiotoxicity and neurotoxicity [2,3]. Levobupivacaine, the pure S(−) enantiomer of bupivacaine, is a safer choice for regional anesthesia than the racemic mixture. Owing to its favorable pharmacologic profile, levobupivacaine has been shown to be as effective as bupivacaine in clinical anesthetic practice in recent years.

Levobupivacaine has been shown to be clinically effective for regional anesthesia following both single-dose epidural administration and continuous postoperative epidural infusion. The incidence of adverse drug reactions is minimal when administered within the recommended dosage range [4]. Ropivacaine, the pure S(−) enantiomer of a long-acting amide local anesthetic, has a structure similar to that of bupivacaine. It is safer than racemic bupivacaine because of its lower potential for cardiotoxicity and neurotoxicity [5]. When regional anesthetic techniques require large volumes of local anesthetic infiltration, ropivacaine offers a clear advantage over bupivacaine because of its lower potential for toxicity.

Compared with intrathecal bupivacaine, intrathecal ropivacaine produces less intense motor block while providing a comparable duration of sensory block because it blocks Aδ and C nerve fibers more intensely than motor fibers (Aβ fibers). The ideal spinal anesthetic agent for ambulatory surgery should provide rapid onset of reliable surgical anesthesia of adequate duration, prompt recovery of sensory and motor function, and minimal complications. Thus, ropivacaine may be well suited for this purpose [5,6]. Therefore, this study was planned to compare the efficacy of intrathecal isobaric 0.75% ropivacaine and isobaric 0.5% levobupivacaine in elective lower abdominal and lower limb surgeries. The primary objective of the present study was to determine the onset and duration of sensory and motor blockade with each drug, and the secondary objective was to compare the Visual Analog Scale (VAS) score and the need for rescue analgesia between the two groups. We aimed to use the findings of this study to help establish a standard of care at our hospital for selecting the optimal anesthetic agent with a lower toxicity profile for infraumbilical surgeries.

This work was previously presented as a paper at the 2020 Indian Society of Anaesthesiologists (ISA) Puducherry State Conference, held on August 6, 2020.

## Materials and methods

Study design and ethical approval

This study was planned as a prospective, randomized, double-blind study. Ninety patients were recruited into this trial after approval by the Institutional Review Board, Sri Venkateshwaraa Medical College Hospital and Research Centre (approval SVMCH/IEC/2019-Nov/21). The study was also registered prospectively with the Clinical Trials Registry- India (CTRI) (CTRI registration no. CTRI/2020/02/023473).

Study setting and participants

We included patients with American Society of Anesthesiologists (ASA) physical status I or II, aged 18-60 years, of either sex, who were scheduled for elective surgeries involving the infraumbilical region under spinal anesthesia at Sri Venkateshwaraa Medical College Hospital and Research Centre, Puducherry, India, between February 2020 and July 2021. Patients with contraindications to spinal anesthesia, including coagulopathies, infection at the injection site, patient refusal, mental illness, pregnancy, or allergy to local anesthetics, were excluded.

Randomization and blinding

Participants were randomized to receive either ropivacaine (Group R) or levobupivacaine (Group L) using a computer-generated block randomization sequence, and allocation was performed using a serially numbered, opaque, sealed-envelope approach. The subarachnoid block was performed by an experienced anesthesiologist who was not involved in the subsequent study procedures or data collection. Both the patients and the anesthesiologist responsible for data collection were blinded to group allocation. Subjects in Group R received 3 mL of 0.75% ropivacaine (7.5 mg/mL; 22.5 mg of ROPIN^®^ 0.75%, Neon Laboratories Ltd., Mumbai, India), whereas those in Group L received 3 mL of 0.5% levobupivacaine (15 mg of LEVO-ANAWIN^®^ 0.5%, Neon Laboratories Ltd.).

Anesthetic procedure and perioperative management

On the night before surgery, all participants received a tablet of metoclopramide 10 mg and a tablet of alprazolam 0.25 mg. On the day of surgery, a large-bore IV cannula was inserted for all patients, and baseline vital signs (blood pressure, pulse rate, SpO₂, ECG, and temperature) were recorded. Patients received coloading with Ringer’s lactate solution during administration of the subarachnoid block. Replacement fluids were administered according to body weight and intraoperative fluid losses. After randomization, the principal investigator provided the sealed envelope to the anesthesiologist responsible for administering spinal anesthesia and providing standard medical care.

The primary investigator evaluated all study parameters for each patient. Spinal anesthesia was performed using a 27-G Quincke-type spinal needle at the L3-L4 or L4-L5 intervertebral space under aseptic precautions. After confirmation of free cerebrospinal fluid flow through the spinal needle, the study drug was administered, and patients were immediately placed in the supine position. Completion of the injection was designated as “zero time” for induction of anesthesia. The onset of sensory block was assessed using a pinprick test or spirit swab every one minute until the T6 dermatomal level was achieved, and surgery was initiated after an adequate level of block was confirmed. Failure was defined as failure to achieve the T10 sensory level within 30 minutes. These patients received general anesthesia. Such cases were excluded from the analysis, and only the total number of failures was reported in the per-protocol analysis. The onset of motor block was assessed using the Modified Bromage Scale [7]. Failure to achieve Grade 3 motor block within 30 minutes was considered a failed block, and these patients were excluded from the study.

Vital signs, including pulse rate, systolic and diastolic blood pressure, mean arterial pressure (MAP), respiratory rate, and oxygen saturation (SpO₂), were monitored for 24 hours following induction of spinal anesthesia at regular intervals during the intraoperative and postoperative periods. Hypotension, defined as a MAP <60 mmHg or a reduction of >20% from baseline blood pressure, was treated with IV mephentermine 6 mg and an additional 100 mL fluid bolus. Bradycardia, defined as a heart rate <50 beats/min or a decrease of >20% from baseline, was treated with IV atropine 0.6 mg. A decrease in SpO₂ below 95% was treated with 100% oxygen at 6 L/min administered via an anatomical face mask with a closed circuit, as required.

Outcome measures

The primary outcome of the study was the comparison of the onset and duration of sensory and motor blockade between the two groups. The onset of sensory block was assessed every five minutes for 30 minutes until the T6 dermatomal level was achieved, and the duration of sensory and motor block was assessed postoperatively for up to 24 hours. The secondary outcome was the comparison of the VAS score and the requirement for rescue analgesia between the groups. The VAS (marked on a continuous 10-cm line, where 0 indicated no pain and 10 indicated the worst possible pain) was assessed at 30-minute intervals from 60 to 300 minutes or until rescue analgesia was administered [8]. Rescue analgesia consisted of IV tramadol 50 mg administered when patients reported a VAS score >4. The total requirement for rescue analgesia was documented. Secondary outcomes, including hemodynamic changes and adverse reactions such as nausea, vomiting, pruritus, and shivering, were also recorded.

Sample size calculation

The sample size was calculated based on the primary outcome variable, duration of postoperative analgesia, using data from the study by Athar et al. comparing intrathecal levobupivacaine and ropivacaine [9]. Assuming a two-sided alpha error of 5% (α = 0.05), a study power of 80% (β = 0.20), and the expected difference in the primary outcome derived from the previous study, the required sample size was estimated to be 45 patients in each group (total sample size = 90). During the study, one patient in the ropivacaine group experienced failed spinal anesthesia requiring conversion to general anesthesia and was therefore excluded from the per-protocol analysis. Consequently, the final analysis included 89 patients (Group L = 45; Group R = 44).

Statistical analysis

Data were analyzed using IBM SPSS Statistics for Windows, version 23.0 (released 2015; IBM Corp., Armonk, NY, USA). Microsoft Excel 2013 (Microsoft Corporation, Redmond, WA, USA) was used to create charts and diagrams. Continuous variables were expressed as mean ± SD, and categorical variables as frequency and percentage. Intergroup comparisons of continuous variables were performed using the independent Student’s t-test or the Mann-Whitney U test, as appropriate. Categorical variables were analyzed using the chi-square test or Fisher’s exact test. Because hemodynamic variables (heart rate, systolic blood pressure, diastolic blood pressure, and MAP) were measured repeatedly over time, they were additionally analyzed using repeated-measures ANOVA with Greenhouse-Geisser correction when the assumption of sphericity was violated. The analysis evaluated the effects of time, treatment group, and the time × group interaction. Bonferroni adjustment was used for pairwise comparisons where applicable. A p-value <0.05 was considered statistically significant.

## Results

**Figure 1:**
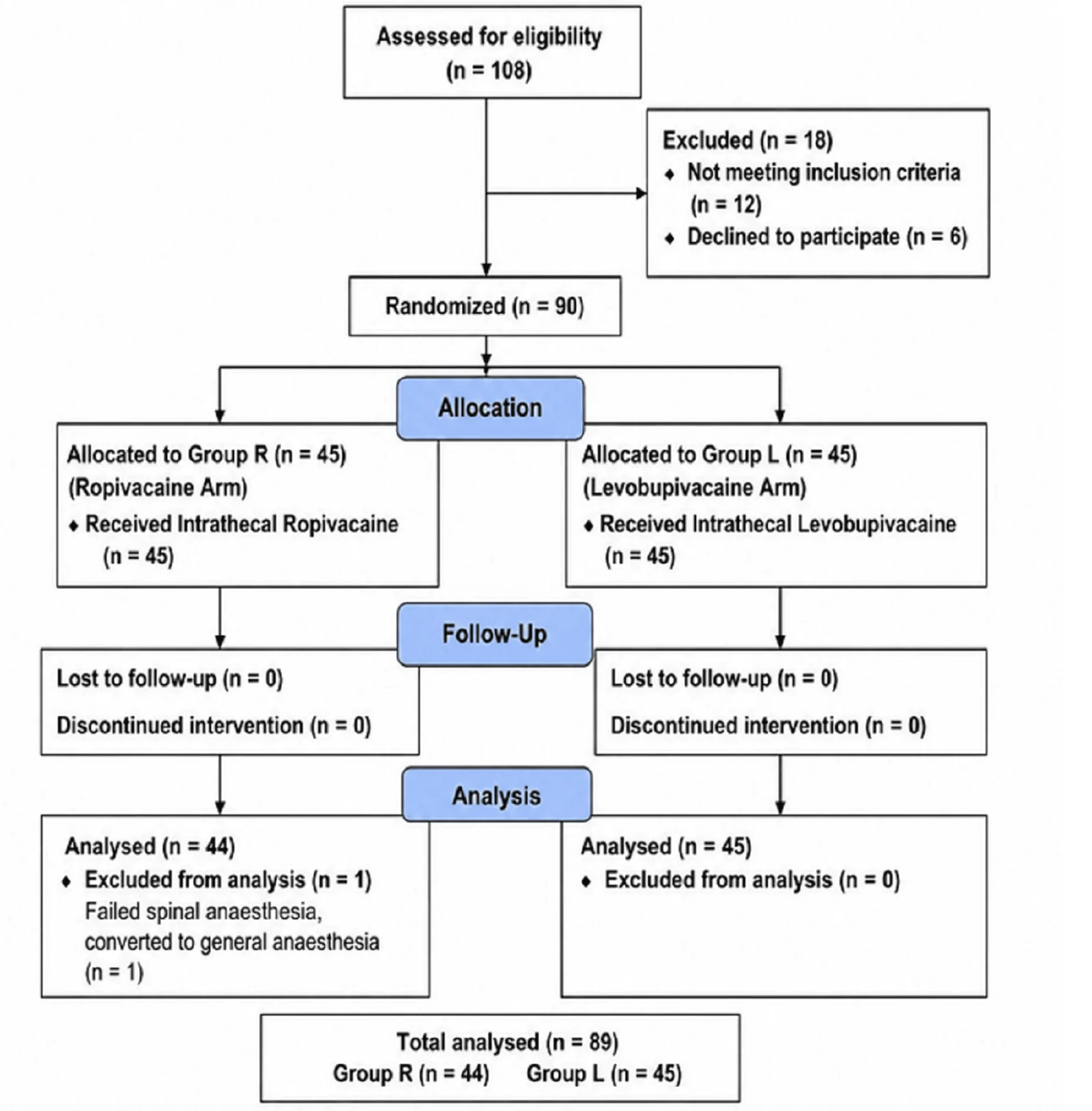
CONSORT flow diagram of the study. CONSORT flow diagram of the study showing that there was no loss to follow-up and that a total of 45 patients in each group were analyzed. CONSORT, Consolidated Standards of Reporting Trials

The Consolidated Standards of Reporting Trials (CONSORT) flow diagram of the study is shown in Figure 1.

The baseline demographic and clinical characteristics of the study population are presented in Table 1.

**Table 1:** Baseline demographic characteristics of the study participants. ASA, American Society of Anesthesiologists

Characteristic		Group L (N = 45)	Group R (N = 44)	Total
Gender	Male	25 (55.6%)	17 (38.6%)	42 (47.2%)
	Female	20 (44.4%)	27 (61.4%)	47 (52.8%)
ASA	I	29 (64.4%)	21 (47.7%)	50 (56.2%)
	II	16 (35.6%)	23 (52.3%)	39 (43.8%)
Age group	18-20 years	4 (8.9%)	3 (6.8%)	7 (7.9%)
	21-30 years	8 (17.8%)	6 (13.6%)	14 (15.7%)
	31-40 years	9 (20%)	9 (20.5%)	18 (20.2%)
	41-50 years	15 (33.3%)	15 (34.1%)	30 (33.7%)
	51-60 years	9 (20%)	11 (25%)	20 (22.5%)

Table 2 compares the sex distribution, age group distribution, ASA physical status, and body weight between the two groups. The majority of patients in both groups were female. There was no statistically significant difference in sex distribution between the two groups (p > 0.05). Patients were categorized into five age groups (18-20, 21-30, 31-40, 41-50, and 51-60 years). The distribution of patients across the age groups was comparable between the two groups, with no statistically significant difference (p > 0.05). The study population comprised adults aged 18-60 years. The majority of patients were classified as ASA physical status I, followed by ASA physical status II. The distribution of ASA physical status was comparable between the two groups, with no statistically significant difference (p > 0.05). The mean body weight was also comparable between Group L and Group R, with no statistically significant difference (p > 0.05).

**Table 2:** Comparison of age group, sex, and ASA physical status between the two groups. ^#^ Not statistically significant (p > 0.05) by the chi-square test. ASA, American Society of Anesthesiologists

Characteristic		Group L (N = 45), n (%)	Group R (N = 44), n (%)	p-value
Gender	Male	25 (55.6)	17 (38.6)	0.14^#^ (χ² = 2.55)
	Female	20 (44.4)	27 (61.4)	
ASA	I	29 (64.4)	21 (47.7)	0.14^#^ (χ² = 2.55)
	II	16 (35.6)	23 (52.3)	
Age group	18-20 years	4 (8.9)	3 (6.8)	0.961^#^ (χ² = 0.617)
	21-30 years	8 (17.8)	6 (13.6)	
	31-40 years	9 (20.0)	9 (20.5)	
	41-50 years	15 (33.3)	15 (34.1)	
	51-60 years	9 (20.0)	11 (25.0)	

The primary outcome measures are presented in Table 3. The mean onset of sensory block was significantly faster in Group L (5.98 ± 1.39 minutes) than in Group R (8.31 ± 1.01 minutes). The difference between the two groups was highly statistically significant (p < 0.001), indicating earlier establishment of sensory blockade with intrathecal levobupivacaine. Group L also demonstrated a significantly faster onset of motor block (8.68 ± 1.81 minutes) than Group R (11.76 ± 1.42 minutes). The difference was highly statistically significant (p < 0.001), suggesting a more rapid onset of motor blockade with levobupivacaine. The mean duration of sensory block was significantly longer in Group L (268.11 ± 42.90 minutes) than in Group R (160.48 ± 51.74 minutes). The difference was highly statistically significant (p < 0.001), demonstrating prolonged sensory anesthesia with levobupivacaine. Similarly, the mean duration of motor block was significantly longer in Group L (230.89 ± 48.42 minutes) than in Group R (136.02 ± 41.62 minutes). The difference was highly statistically significant (p < 0.001), indicating prolonged motor blockade with levobupivacaine. The duration of postoperative analgesia was also significantly longer in Group L (275.22 ± 39.54 minutes) than in Group R (207.66 ± 39.34 minutes). The difference was highly statistically significant (p < 0.001), reflecting superior and prolonged postoperative analgesia with levobupivacaine.

**Table 3:** Comparison of block characteristics between the two groups. ^*^ Highly statistically significant at p < 0.001 by the independent-samples t-test.

Outcome measure	Group L (N = 45), mean ± SD	Group R (N = 44), mean ± SD	t-value	p-value
Onset of sensory blockade (minutes)	5.98 ± 1.39	8.31 ± 1.01	9.01	<0.001^*^
Onset of motor blockade (minutes)	8.68 ± 1.81	11.76 ± 1.42	8.92	<0.001^*^
Duration of sensory blockade (minutes)	268.11 ± 42.90	160.48 ± 51.74	10.34	<0.001^*^
Duration of motor blockade (minutes)	230.89 ± 48.42	136.02 ± 41.62	9.9	<0.001^*^
Duration of analgesia (minutes)	275.22 ± 39.54	207.66 ± 39.34	8.08	<0.001^*^

**Figure 2:**
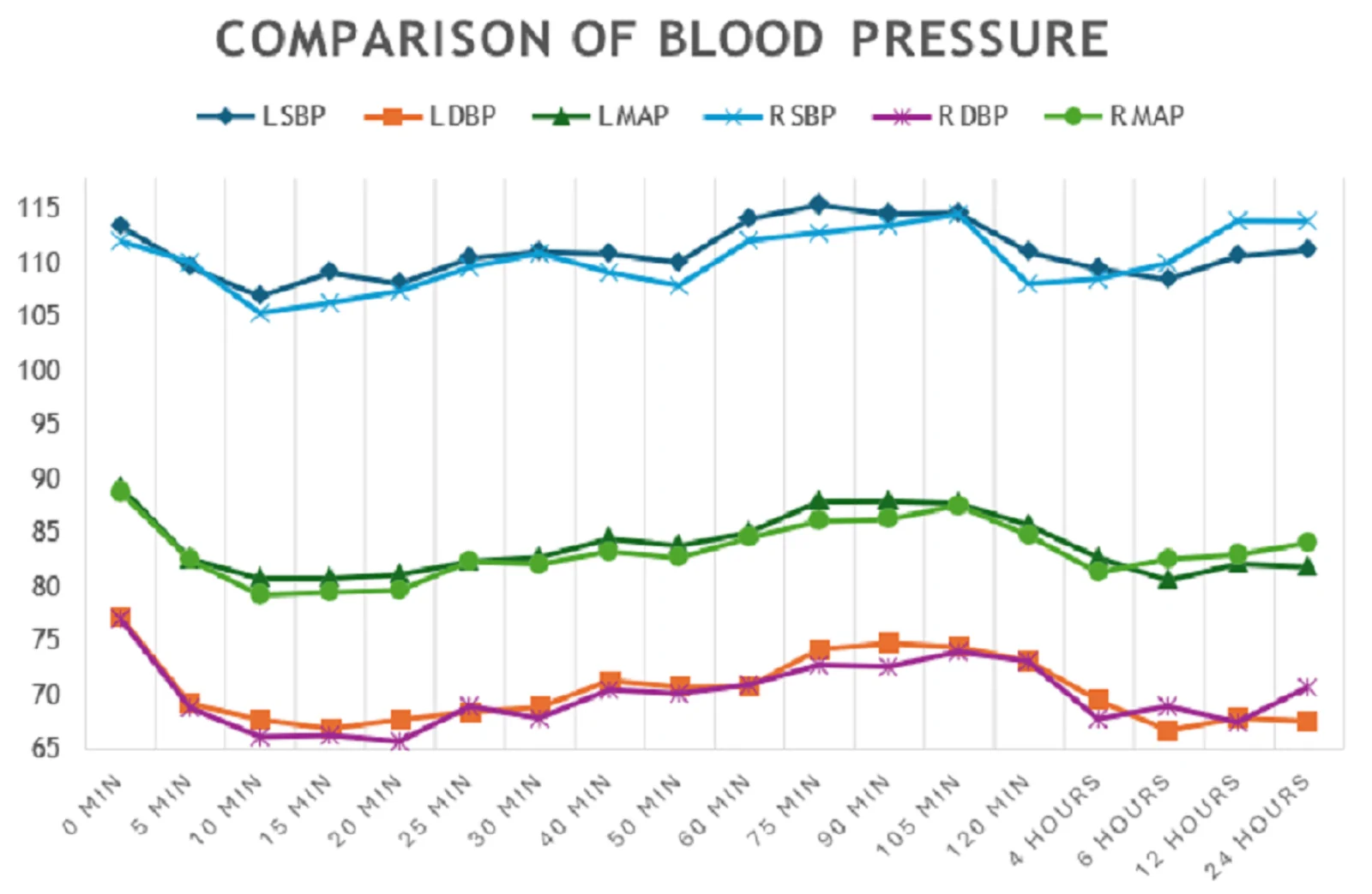
Comparison of SBP, DBP, and MAP between the two groups. DBP, diastolic blood pressure; LDBP, left diastolic blood pressure; LMAP, left mean arterial pressure; LSBP, left systolic blood pressure; MAP, mean arterial pressure; RDBP, right diastolic blood pressure; RMAP, right mean arterial pressure; RSBP, right systolic blood pressure; SBP, systolic blood pressure

**Figure 3:**
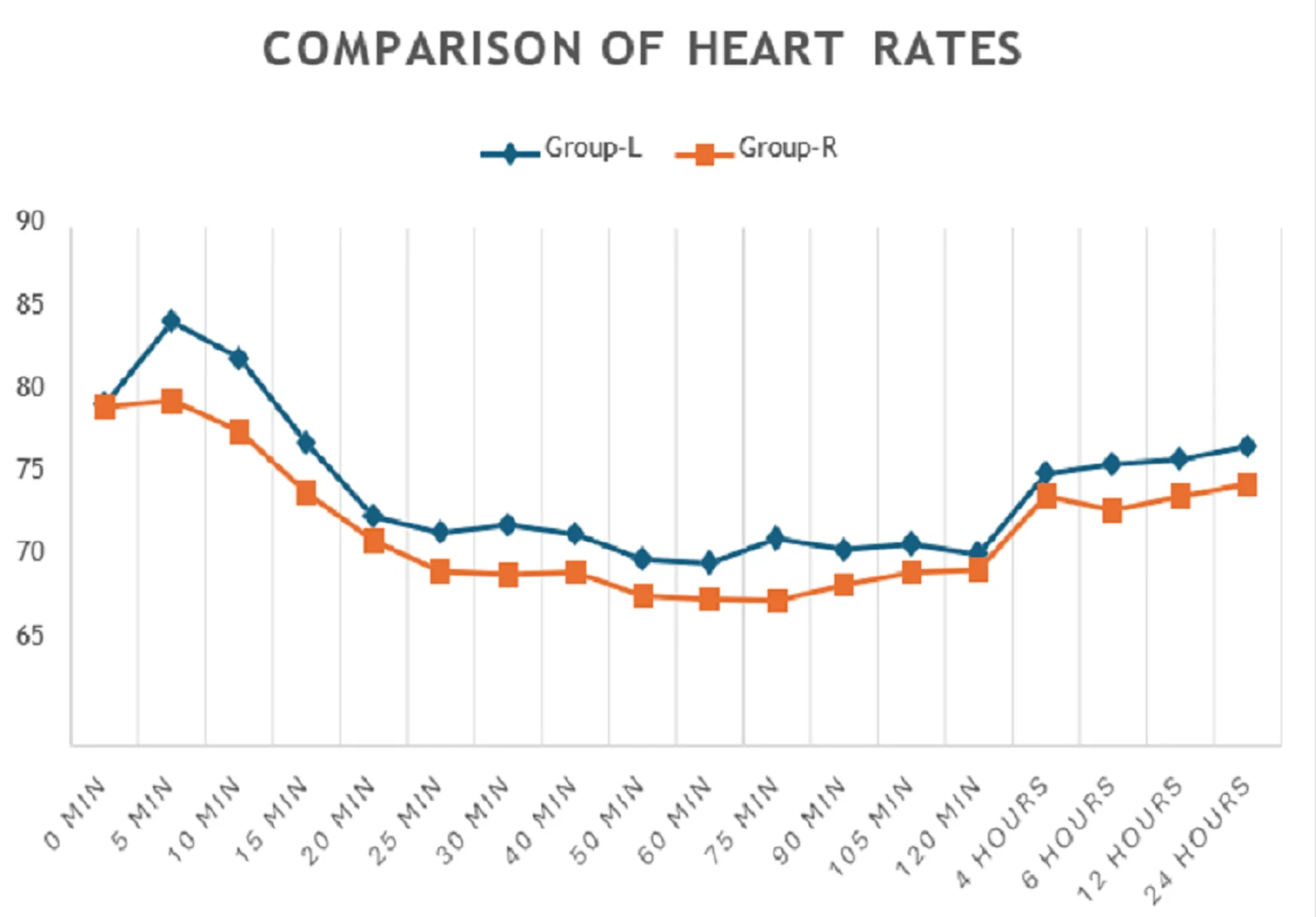
Comparison of heart rate between Groups L and R.

Comparisons of systolic blood pressure, diastolic blood pressure, MAP, and heart rate between the two groups are shown in Figure 2 and Figure 3.

Systolic blood pressure remained comparable between the two groups throughout the intraoperative and postoperative periods. Repeated-measures ANOVA with Greenhouse-Geisser correction demonstrated a significant effect of time on systolic blood pressure (F = 8.065, p < 0.001), indicating expected perioperative changes over time. However, there was no significant overall group effect (F = 0.295, p = 0.588) or time × group interaction (F = 1.052, p = 0.393), suggesting that both levobupivacaine and ropivacaine maintained comparable systolic blood pressure profiles during the study period.

Diastolic blood pressure remained comparable between the two groups throughout the intraoperative and postoperative periods. Repeated-measures ANOVA with Greenhouse-Geisser correction demonstrated a significant effect of time on diastolic blood pressure (F = 21.363, p < 0.001), indicating expected perioperative changes over time. However, there was no significant overall group effect (F = 0.140, p = 0.709) or time × group interaction (F = 1.075, p = 0.379), suggesting that both levobupivacaine and ropivacaine maintained comparable diastolic blood pressure profiles during the study period.

MAP remained stable and comparable between the two groups throughout the intraoperative and postoperative periods. Repeated-measures ANOVA with Greenhouse-Geisser correction demonstrated a significant effect of time on MAP (F = 21.847, p < 0.001), indicating expected perioperative changes over time. However, there was no significant overall group effect (F = 0.240, p = 0.626) or time × group interaction (F = 1.063, p = 0.385), suggesting that both levobupivacaine and ropivacaine maintained comparable MAP profiles during the study period.

Heart rate remained comparable between the two groups throughout the intraoperative and postoperative periods. Repeated-measures ANOVA with Greenhouse-Geisser correction demonstrated a significant effect of time on heart rate (F = 29.209, p < 0.001), indicating expected perioperative changes over time. However, there was no significant overall group effect (F = 1.379, p = 0.244) or time × group interaction (F = 0.562, p = 0.705), suggesting that both levobupivacaine and ropivacaine maintained comparable heart rate profiles during the study period.

The comparison of VAS scores is presented in Table 4. The VAS was assessed at 30-minute intervals using a 0-10 scale. Postoperative VAS scores were consistently lower in Group L than in Group R during the early postoperative period, indicating superior analgesia with levobupivacaine. The intergroup difference at the 12-hour postoperative time point was statistically significant (p = 0.017).

**Table 4:** Comparison of VAS scores between Groups L and R. ^*^ Statistically significant at p < 0.05 by the Mann-Whitney U test. ^#^ Not statistically significant (p > 0.05) by the independent-samples t-test. VAS, Visual Analog Scale

Time point	Group L (N = 45), mean ± SD	Group R (N = 44), mean ± SD	z-value	p-value
Four hours	0.49 ± 0.506	0.61 ± 0.579	0.942	0.346^#^
Six hours	1.13 ± 0.661	1.18 ± 0.691	0.093	0.926^#^
12 hours	0.42 ± 0.499	0.70 ± 0.553	2.38	0.017^*^
24 hours	0.11 ± 0.318	0.14 ± 0.347	0.36	0.719^#^

**Figure 4:**
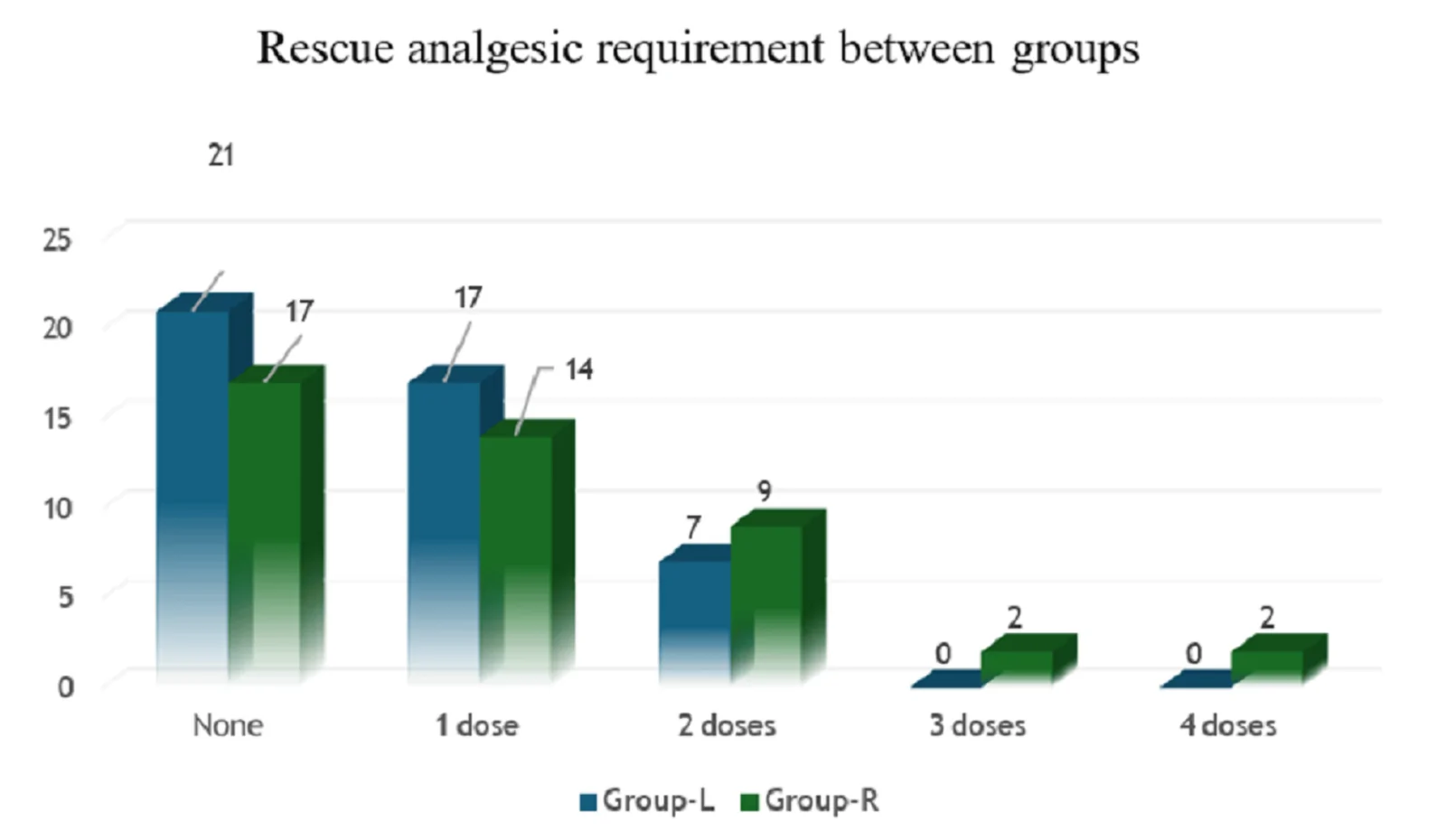
Comparison of postoperative rescue analgesic dose requirements between Groups L and R.

The comparison of postoperative rescue analgesic requirements between the two groups is presented in Table 5 and Figure 4. Although patients in Group L required fewer postoperative rescue analgesic doses than those in Group R, the difference was not statistically significant, either in the distribution of rescue analgesic doses (chi-square test, p = 0.292) or in the mean number of rescue analgesic doses (p = 0.075).

**Table 5:** Comparison of postoperative rescue analgesic requirements between Groups L and R. ^#^ Not statistically significant (p > 0.05) by the chi-square test. ^†^ Not statistically significant (p > 0.05) by the independent-samples t-test.

Number of rescue analgesic doses	Group L (N = 45), n (%)	Group R (N = 44), n (%)	p-value
None	21 (46.7)	17 (38.6)	0.292^#^ (χ² = 4.95)
One dose	17 (37.8)	14 (31.8)	
Two doses	7 (15.6)	9 (20.5)	
Three doses	0 (0.0)	2 (4.5)	
Four doses	0 (0.0)	2 (4.5)	
Mean ± SD	0.69 ± 0.73	1.05 ± 1.10	0.075^†^ (t = 1.8)

## Discussion

Compared with ropivacaine, our study demonstrated that isobaric levobupivacaine provided a longer duration of analgesia with a more rapid onset of sensory and motor blockade. The mean onset of sensory block was significantly faster in Group L (5.98 ± 1.39 minutes) than in Group R (8.31 ± 1.01 minutes). The difference between the two groups was highly statistically significant (p < 0.001), indicating earlier establishment of sensory blockade with intrathecal levobupivacaine. Group L also demonstrated a significantly faster onset of motor block (8.68 ± 1.81 minutes) compared with Group R (11.76 ± 1.42 minutes). The difference was highly statistically significant (p < 0.001), suggesting a more rapid onset of motor blockade with levobupivacaine. The mean duration of sensory block was significantly longer in Group L (268.11 ± 42.9 minutes) than in Group R (160.48 ± 51.74 minutes). The difference was highly statistically significant (p < 0.001), demonstrating prolonged sensory anesthesia with levobupivacaine. The mean duration of motor block was significantly longer in Group L (230.89 ± 48.42 minutes) compared with Group R (136.02 ± 41.62 minutes). The difference was highly statistically significant (p < 0.001), indicating prolonged motor blockade with levobupivacaine. The duration of postoperative analgesia was significantly longer in Group L (275.22 ± 39.54 minutes) than in Group R (207.66 ± 39.34 minutes). The difference was highly statistically significant (p < 0.001), reflecting superior and prolonged postoperative analgesia with levobupivacaine.

Baseline hemodynamic parameters and patient characteristics did not differ significantly between the two groups. No significant adverse effects were observed in either group. Levobupivacaine and ropivacaine are two contemporary amide local anesthetics used to reduce the toxicity associated with bupivacaine [10,11]. Several studies by Lee et al., Kannai et al., and Gautier et al. have demonstrated that levobupivacaine has efficacy comparable to that of bupivacaine and ropivacaine while exhibiting an improved safety profile [11-13]. Numerous studies have compared their clinical efficacy, baricity, and potency at varying doses.

Lee et al. conducted a study comparing bupivacaine, levobupivacaine, and ropivacaine and concluded that levobupivacaine and bupivacaine are equipotent, whereas ropivacaine is only two-thirds as potent as the other two drugs [14]. The greater lipid solubility of levobupivacaine may partially account for its higher potency compared with ropivacaine [15]. Several studies have demonstrated that levobupivacaine has approximately 30% greater potency than ropivacaine [16,17]. Consequently, based on previous studies, we selected concentrations of 5 mg/mL for levobupivacaine and 7.5 mg/mL for ropivacaine while maintaining a constant volume.

Samar et al. reported that the onset of sensory blockade in the levobupivacaine group was significantly faster (6.97 ± 1.82 minutes) than in the ropivacaine group (8.47 ± 2.55 minutes) (p < 0.05) [18]. They also observed that the onset of motor block was 10.27 ± 1.92 minutes in the levobupivacaine group compared with 12.93 ± 2.55 minutes in the ropivacaine group (p < 0.05). Similarly, Patel et al. compared levobupivacaine and ropivacaine for spinal anesthesia and found that the mean onset of sensory block was 4.74 ± 0.828 minutes in the levobupivacaine group and 6.47 ± 0.861 minutes in the ropivacaine group (p < 0.001) [19]. These findings are consistent with those of our study. They also reported that the time to achieve Modified Bromage Scale grade 3 was 6.68 ± 1.147 minutes in the levobupivacaine group and 7.97 ± 0.87 minutes in the ropivacaine group (p < 0.001).

Athar et al., in a study comparing equivalent intrathecal doses of levobupivacaine and ropivacaine, found that the onset of sensory blockade was significantly shorter in the ropivacaine group (13.17 ± 3.02 minutes) than in the levobupivacaine group (20.33 ± 5.31 minutes; p < 0.0001), which contradicts our findings [9]. They also reported that the time to achieve Modified Bromage Scale grade 3 was shorter in the ropivacaine group (7.83 ± 2.84 minutes) than in the levobupivacaine group (12.17 ± 4.09 minutes; p < 0.0001), a finding that differed from previous studies. Our results are therefore not consistent with those of Athar et al., as we observed a faster onset of motor blockade in Group L than in Group R. This discrepancy may be attributable to differences in demographic characteristics, baricity, methodology, or drug dosage. However, apart from the faster onset of sensory block, the other outcomes reported by Athar et al., including the duration of sensory blockade and postoperative analgesia, were longer in the levobupivacaine group than in the ropivacaine group [9]. Govindarao et al. also demonstrated that motor block developed significantly more slowly in patients receiving intrathecal ropivacaine (12.63 ± 3.5 minutes) than in those receiving levobupivacaine (9.03 ± 2.67 minutes) (p = 0.000) [20]. Mantouvalou et al. compared plain ropivacaine, bupivacaine, and levobupivacaine for lower abdominal surgery and concluded that the onset of motor block was significantly earlier in patients receiving bupivacaine than in those receiving ropivacaine and was comparable to that in the levobupivacaine group (p < 0.05) [21].

Several studies have attributed the greater potency of levobupivacaine to its higher lipid solubility compared with ropivacaine [17,18,21,22]. The greater lipid solubility and protein binding reported in previous studies may explain the longer duration of sensory and motor blockade observed in the levobupivacaine group in our study. This suggests that patients in Group L experienced a longer duration of analgesia than those in Group R. The longer duration of sensory blockade is clinically important because it may reduce the need for rescue analgesics and potentially decrease postoperative opioid consumption.

A longer duration of postoperative analgesia is desirable because it may reduce postoperative pain-related stress and improve patient satisfaction. However, prolonged motor blockade is less desirable because it may delay mobilization in ambulatory surgery. Therefore, levobupivacaine may be better suited for procedures of longer expected duration. We did not measure the time to ambulation or the quality of recovery.

Limitations

This was a single-center study, and the study population was limited to ASA physical status I and II patients undergoing elective infraumbilical procedures. This limits the generalizability of the findings to higher-risk populations, emergency surgeries, obstetric cases, or patients with significant cardiovascular instability, in whom hemodynamic responses may differ considerably. Maintenance of drug temperature could be a challenge in tropical countries and may have influenced the study results. Postoperative follow-up was limited to 24 hours. Therefore, late complications, prolonged neurological outcomes, and patient-centered recovery parameters, such as time to ambulation, discharge readiness, and long-term analgesic quality, were not evaluated. Institutional practices, anesthetic technique variations, and perioperative care protocols may also limit the generalizability of the findings. Future studies with larger, multicenter designs, inclusion of high-risk populations, and incorporation of objective monitoring modalities are warranted to validate and extend these findings.

## Conclusions

Both ropivacaine and levobupivacaine provided reliable subarachnoid blockade and can be used for infraumbilical surgeries. Intrathecal levobupivacaine produced a longer sensory blockade and postoperative analgesia than ropivacaine but was also associated with a longer duration of motor blockade. These findings suggest a trade-off between prolonged analgesia and delayed motor recovery. Selection of the intrathecal agent should therefore be individualized according to the clinical priorities and surgical requirements. Both agents exhibit favorable cardiovascular safety profiles and serve as safer alternatives to bupivacaine for spinal anesthesia in infraumbilical procedures. The longer duration of motor blockade observed with levobupivacaine may delay ambulation in patients undergoing day-case surgery. Future multicenter studies with larger sample sizes and more diverse surgical populations may further validate these findings and help refine drug selection according to individual patient and procedural needs.
